# Langerhans Cell Histiocytosis of the Mandible

**DOI:** 10.7759/cureus.28222

**Published:** 2022-08-21

**Authors:** Kamalakannan Padmanaban, Arunkumar Kamalakaran, Priyadharshini Raghavan, Triveni Palani, Davidson Rajiah

**Affiliations:** 1 Oral and Maxillofacial Surgery, Tamilnadu Government Dental College and Hospital, Chennai, IND

**Keywords:** immunohistochemistry, radiolucency, mandibular swelling, lytic lesion, eosinophilic granuloma, langerhans cell histiocytosis

## Abstract

The unusual disorder known as Langerhans cell histiocytosis, which is most frequently seen in children and young adults, is caused by the clonal proliferation of Langerhans cells. Even if clinical signs and radiographic evidence of destructive bone lesions may raise suspicion of the disease, a reliable diagnosis without a thorough pathological examination is challenging. This report describes a case of eosinophilic granuloma of the mandible in a nine-year-old child with characteristic radiological, histopathological, and immunohistochemical features.

## Introduction

Histiocytosis collectively refers to a group of uncommon diseases of the reticuloendothelial system. Langerhans cell histiocytosis (LCH) is characterized by aberrant growth of mature eosinophils and specialized bone marrow-derived antigen-presenting dendritic cells [1]. First, to identify the clinical and pathological traits shared by the numerous disease presentations, Lichtenstein recommended that they be categorized as histiocytosis X [1,2]. The word "X" was used to emphasize that there are still many questions about the nature and origin of the disease [1,3]. The three main components of LCH are eosinophilic granuloma, Hand-Schuller-Christian disease, and Letterer-Siwe disease. Eosinophilic granuloma is a localized condition affecting the bone [3]. Eosinophilic granuloma causes 60% to 70% of all cases of LCH and can manifest as solitary (50% to 75%) or multifocal bone abnormalities [4]. The Hand-Schuller-Christian disease is a subacute or chronic condition that is characterized primarily by the triad of "geographical skull" (caused by numerous calvarial lesions), diabetes insipidus, and exophthalmos [3]. The Letterer-Siwe disease is a widespread acute or subacute disease [3]. Nezelof et al. [5], reported in 1973 using the Birbeck granule as a marker that the lesions of histiocytosis X were caused by the growth and dispersion of aberrant histiocytic cells of the Langerhans cell system, leading to the change in nomenclature to Langerhans cell histiocytosis [1,3]. LCH can occur in anywhere between 0.5 and 5.4 cases per million people per year [1]. The incidence in adults is one to two cases per million [6]. People of all ages, from the newborn to the elderly, can develop Langerhans cell histiocytosis; the highest incidence occurs between one and four years [7]. Less than 10% of children with the illness have jaw involvement [8]. Siwe proposed in 1933 that LCH was neither hereditary nor familial. However, there have been numerous reports, including three sets of identical twins, which suggest familial occurrence [9]. This article presents a rare case of unifocal eosinophilic granuloma of the mandible in a nine-year-old boy who presented with a gradually increasing swelling on the left side of his face over one month. We highlight the clinical and microscopic features of this rare case and stress the importance of histopathological examination in the diagnosis of this unusual condition.

## Case presentation

**Figure 1:**
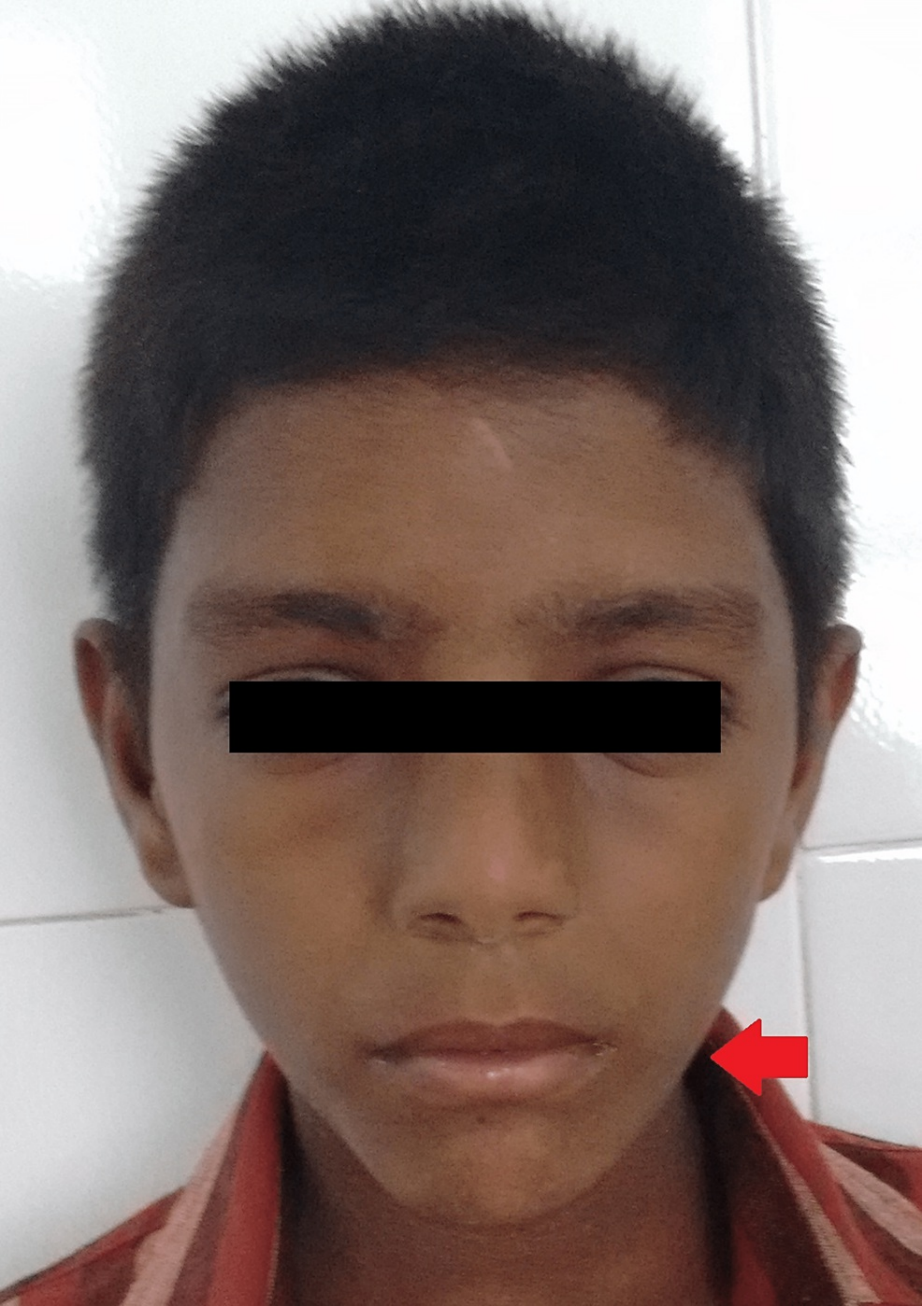
Swelling on the left side of the lower jaw

A nine-year-old male patient reported to the hospital with the complaint of swelling in the left lower jaw for the past one month (Figure 1). Clinical examination revealed a bony hard swelling in relation to the left body of the mandible, 2 X 0.5 cm in size, roughly round to oval in shape, non-tender, with a smooth surface, and ill-defined borders. The skin over the swelling appeared normal. Intraorally, the swelling was obliterating the buccal sulcus in relation to the 75 and 36 regions with grade III mobility of 75. The lesion was negative on aspiration.

**Figure 2:**
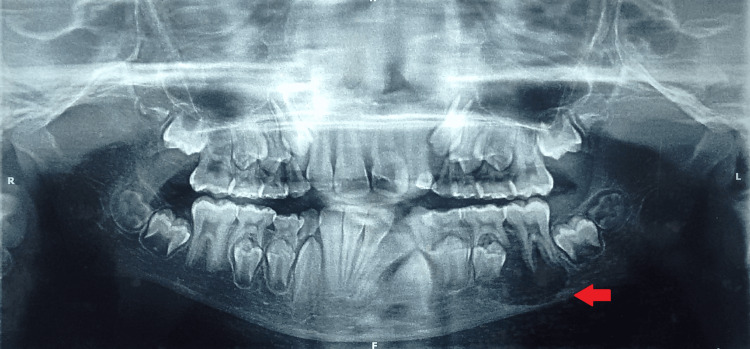
Orthopantamogram showing a well-defined radiolucent lesion in the left body of the mandible region

**Figure 3:**
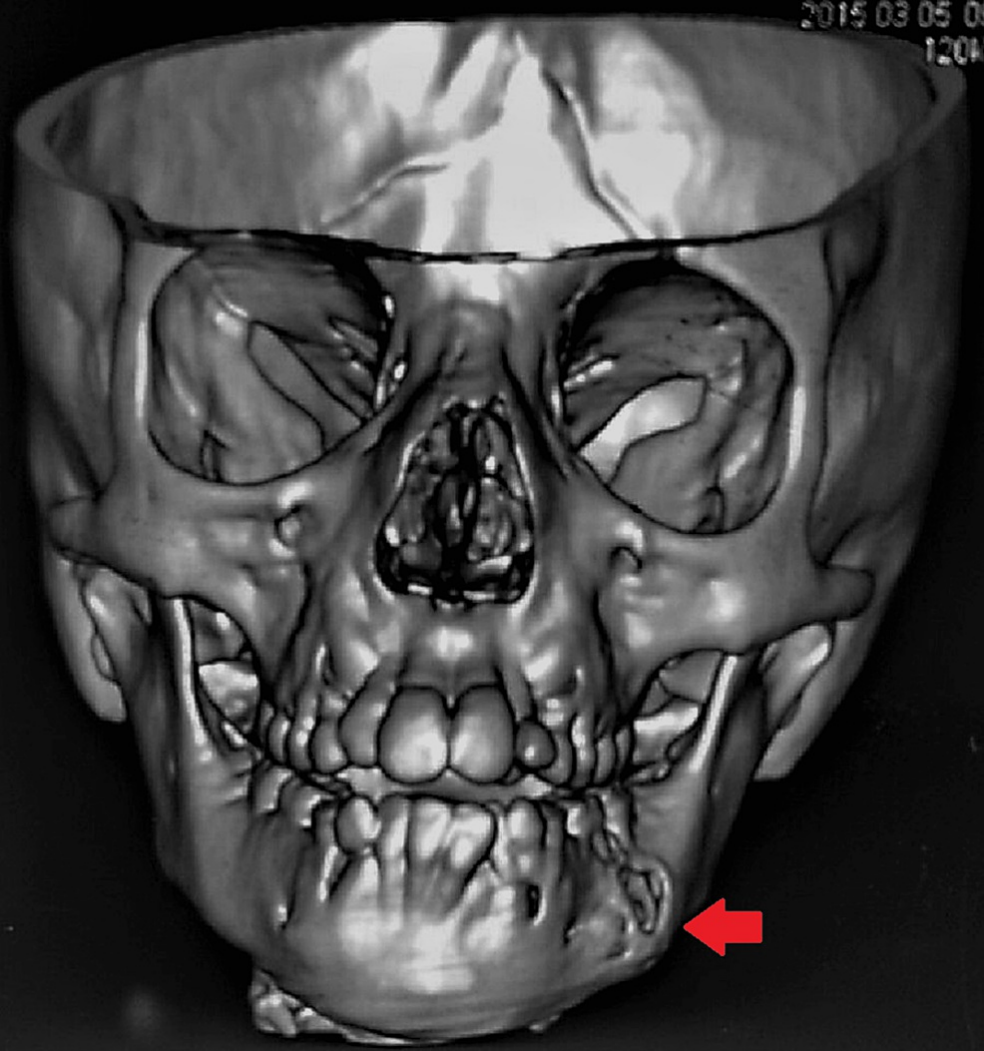
Computed tomography scan of facial bone showing a lytic lesion in the left mandibular region with buccal cortical expansion and perforation

Orthopantamogram revealed a well-defined radiolucent lesion in relation to the left body of the mandible with discrete borders and no evidence of sclerosis. The associated teeth appeared normal (Figure 2). A computed tomography (CT) scan showed a solid lytic lesion in relation to the left lower jaw with both buccal and lingual cortical expansion and perforation (Figure 3). Based on the clinical and radiological findings, the differential diagnosis included ameloblastoma, keratocyst, lymphoma, and eosinophilic granuloma. The increase in volume with cortical bone growth and a radiolucent region typical of bone tissue breakdown often validated the predisposition to suspect these diseases. Furthermore, the involvement of tooth germ leads to the suspicion of the lesion being keratocyst or ameloblastoma.

**Figure 4:**
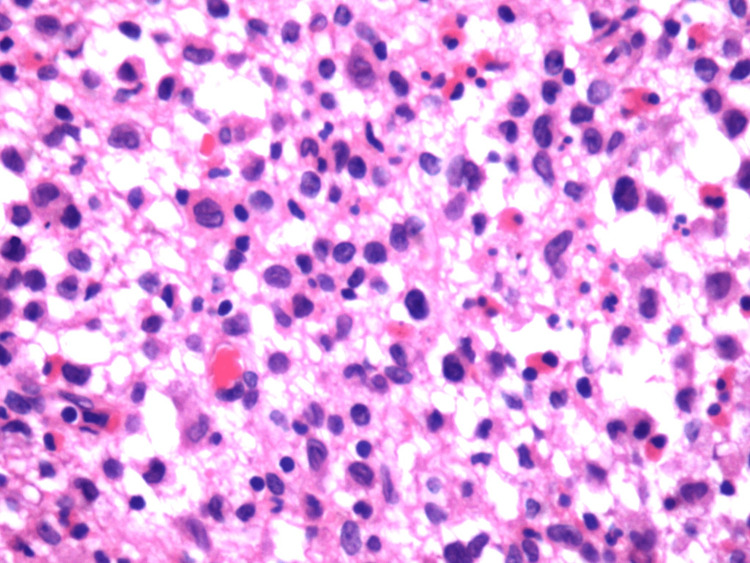
Photomicrograph of the biopsied specimen stained with hematoxylin and eosin

**Figure 5:**
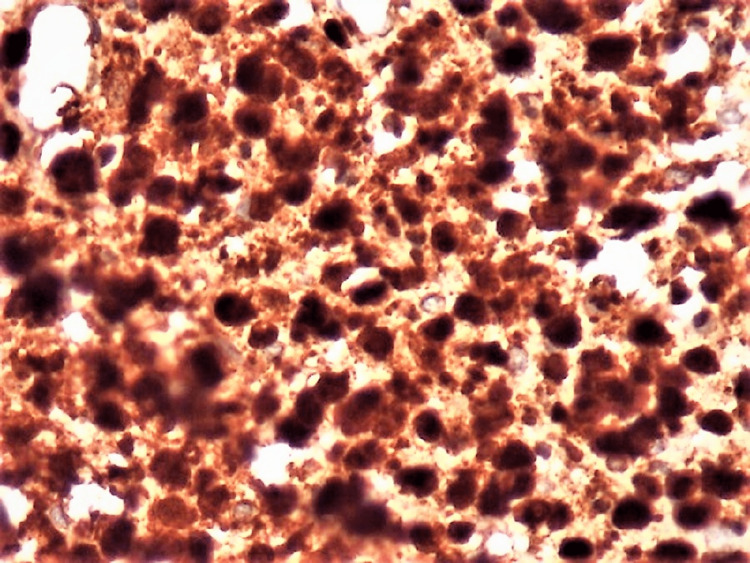
Immunohistochemical staining of the specimen showing positive staining with S100

**Figure 6:**
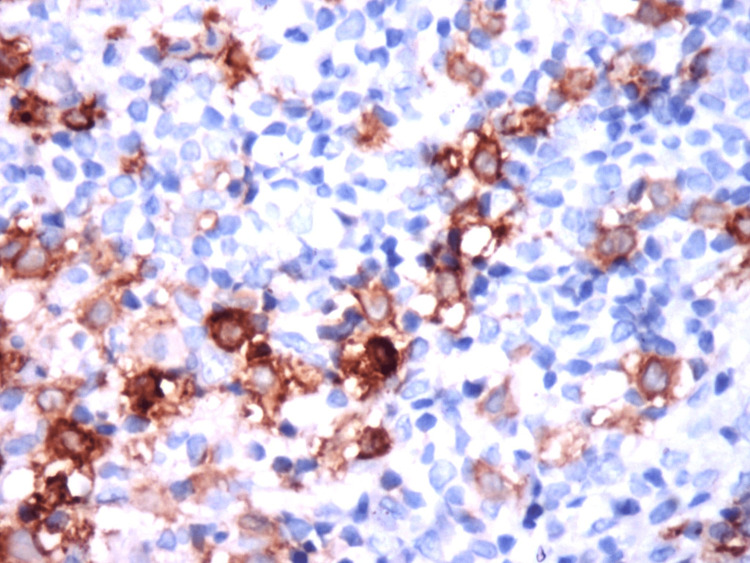
Immunohistochemical staining of the specimen showing positive staining with CD1a

An incisional biopsy was performed under local anesthesia. Histopathological findings of the biopsied specimen revealed hematoxylin-eosin-stained Langerhans cells typically having a moderate amount of homogeneous, pink, granular cytoplasm with distinct cell margins. There was profuse infiltration of eosinophils, plasma cells, and lymphocytes suggestive of Langerhans cell histiocytosis (Figure 4). Further, the biopsied specimen was subjected to immunohistochemical staining and was positive for S100 and CD1a, confirmative of LCH (Figures 5-6). Hence, a systemic skeletal survey was carried out to rule out multifocal involvement, which yielded only negative clues, and thus it was concluded to be a case of restricted/localized Langerhans cell histiocytosis (eosinophilic granuloma of the mandible).

**Figure 7:**
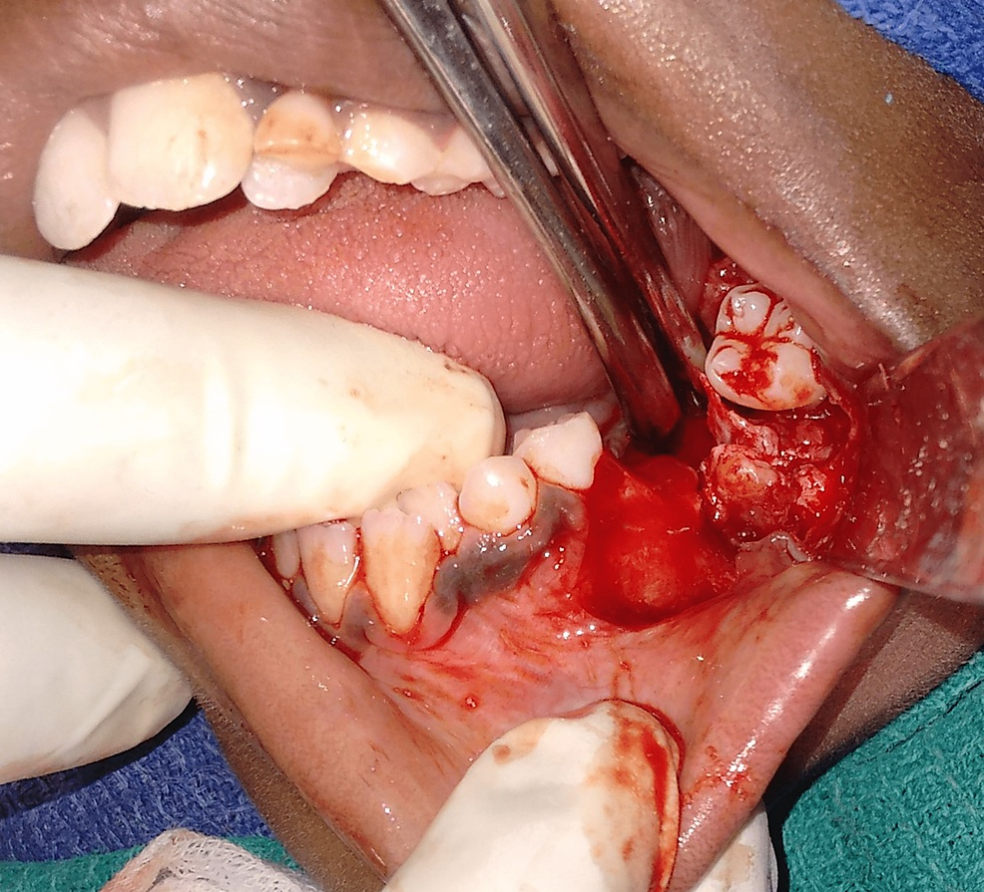
Exposure of the lesion

**Figure 8:**
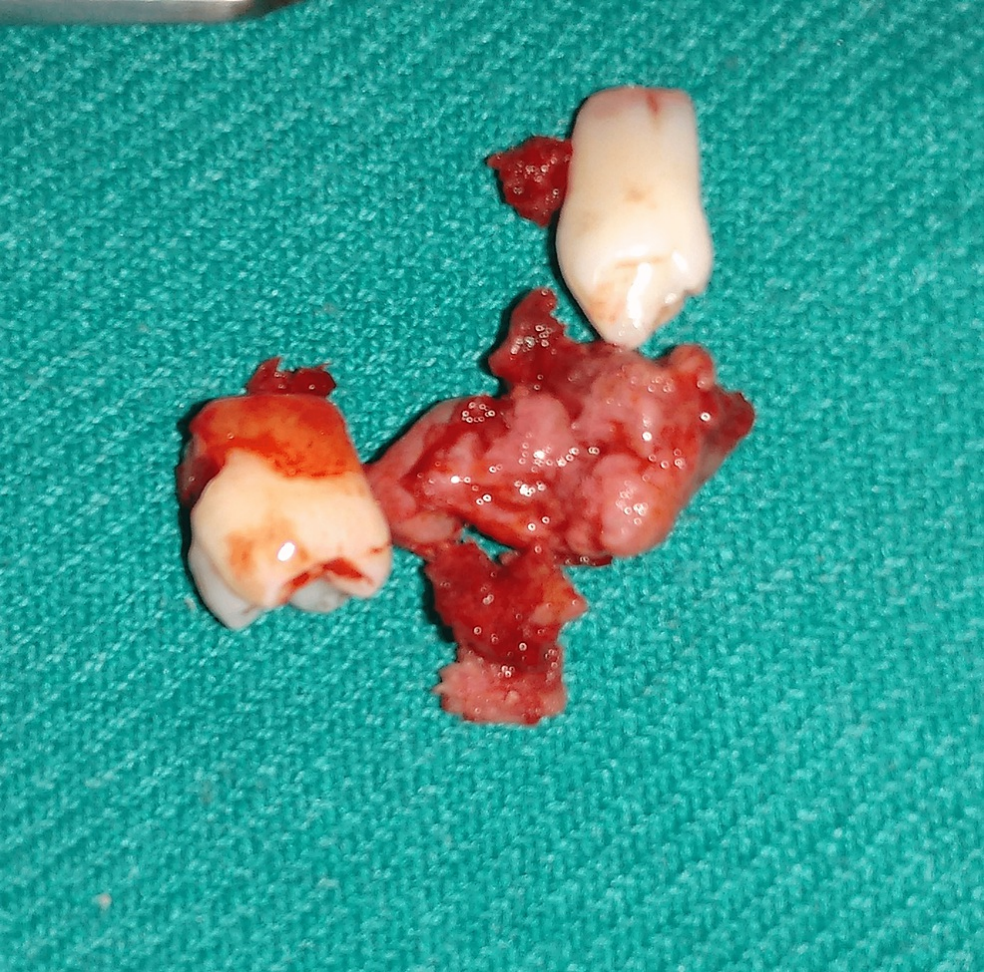
Lesion removed along with associated teeth

**Figure 9:**
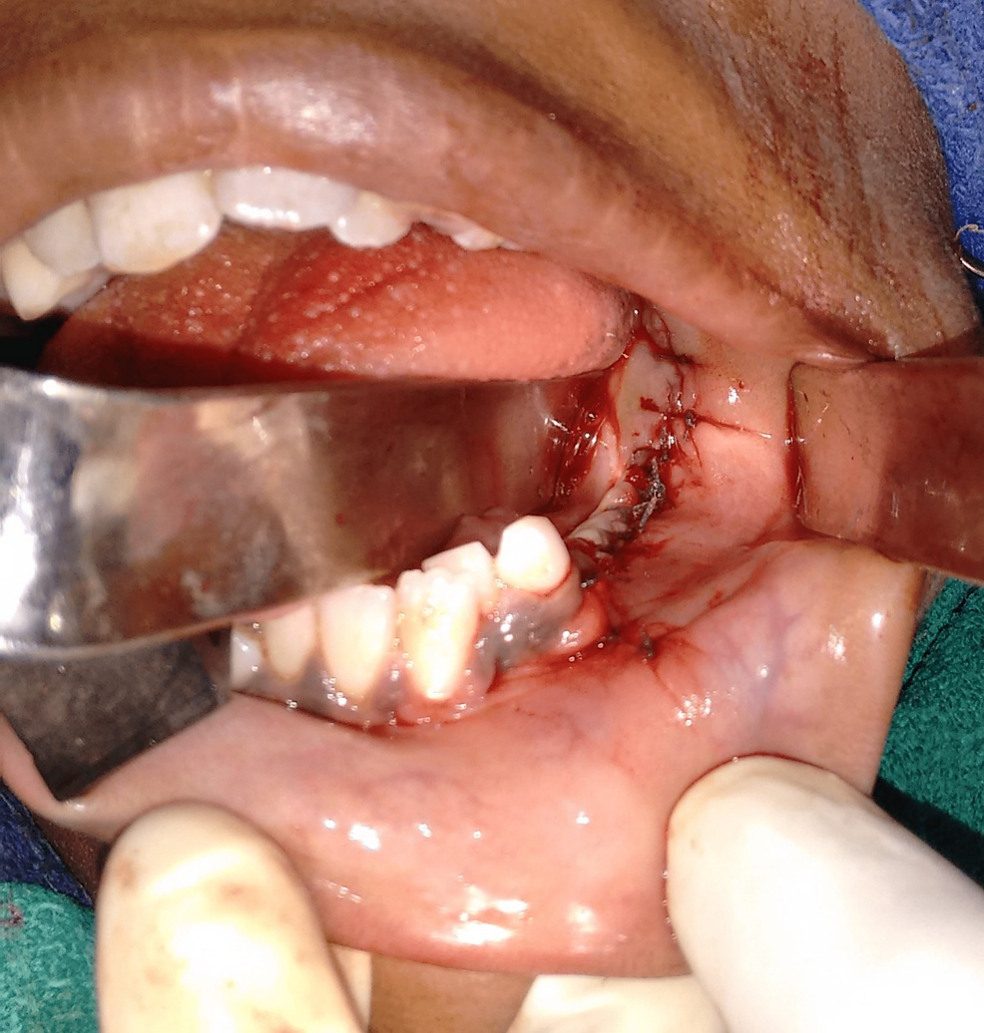
Closure done with 3-0 vicryl sutures

**Figure 10:**
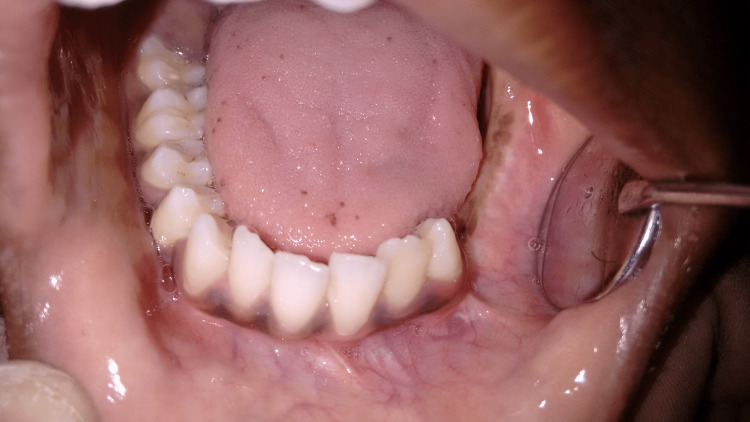
Postoperative healing of the intraoral surgical site

The case was operated on under general anesthesia and the lesion was completely excised with thorough curettage of the surgical bed (Figures 7-9). Excisional biopsy confirmed the preoperative diagnosis. Adequate healing of the intraoral surgical site was observed postoperatively (Figure 10). The patient was followed up regularly for a period of three years and showed no signs of recurrence. Interestingly, the patient had a twin brother, and he was also subjected to clinical evaluation and orthopantomogram, which yielded no positive results.

## Discussion

Histiocytosis is a rare disease of the reticuloendothelial system characterized by the proliferation of specialized bone marrow-derived antigen-presenting dendritic cells [1]. The etiology of Langerhans cell histiocytosis (LCH) is unknown; however, it might be brought on by an infectious antigenic stimulation, a genetic anomaly, a dysregulated immunological response, a cytokine-mediated cellular growth of Langerhans cells, or even clonal origin [1,3]. Granulocyte-macrophage colony-stimulating factor, interleukin-3, and tumor necrosis factor-alpha have all been linked to the potential development of LCH, and more recently, cytogenic studies have proposed the involvement of tumor suppressor genes (p53), oncogenes (c-myc, h-ras), growth factors, cell surface immunologic markers, and apoptotic factors in LCH as well [8]. LCH patients are categorized based on the extent of the disease as restricted Langerhans cell histiocytosis and extensive Langerhans cell histiocytosis [3]. Restricted Langerhans cell histiocytosis* *is characterized by isolated biopsy-proven skin lesions, monostotic lesions (with or without skin rash, lymph node involvement, or diabetes insipidus), or polyostotic lesions (with lesions in multiple bones or more than two lesions in a single bone, with or without skin rash, adjacent lymph node involvement, or diabetes insipidus). Extensive Langerhans cell histiocytosis* *is characterized by the involvement of visceral organs, with or without diabetes insipidus, bone lesions, skin rash, or adjacent lymph node involvement, without signs of dysfunction of the hemopoietic system, lungs, or liver [3].

Langerhans cell histiocytosis can present as anything from a solitary bone lesion that spontaneously regresses to a multisystem, potentially fatal disorder. Multiple organ systems may be involved in the extensive form of LCH, including the bones, skin, lymph nodes, bone marrow, liver, spleen, lungs, gastrointestinal tract, thymus, and endocrine and central nervous systems. Few forms of LCH require little to no treatment while other kinds necessitate aggressive therapy [9]. One hundred fourteen (114) cases (10%) out of the 1120 LCH cases that Hartman studied had oral involvement [10]. Fifty-three percent and 25%, respectively, of the entire group were made up of the monostotic and polyostotic forms of eosinophilic granuloma [4]. Eosinophilic granulomas of the skull have never been reported to occur in families before. Masaru Takahashi and Satoshi Kuwabara presented identical twins with eosinophilic granuloma of the skull [10]. The skull is the bone that is most frequently afflicted, followed by the long bones of the upper extremities and flat bones [11]. A plain radiograph generally shows one or more irregularly marginated lytic lesions of bone. The presentation might resemble mastoiditis when the mastoid process is affected while deafness and ossicle damage result from middle ear extension. In the spine, the lytic process may cause the vertebral body to compress and collapse, leading to vertebra plana [3].

Eosinophilic granuloma of the bone is a rare disease of the maxillofacial region, with an annual incidence rate ranging from one new case in 350000 to 2 million [8]. Around 76% of lesions are found in the mandible, with the mandibular ramus and body being involved in over 96% of cases, according to a recent review of the literature by Guruprasad & Chauhan et al. [12]. These statistics are strikingly similar to those of the case at hand. According to Aricó and Egeler, individuals with eosinophilic granuloma frequently seek consultation due to uncomfortable swelling [13]. There have also been reports of mouth opening limitations and facial asymmetry brought on by the lesion's growth [4]. LCH of the jaws is frequently accompanied by nearby soft tissue edema, "floating" teeth, gingival edema, fractures, or discomfort. Oral symptoms are often limited to a small number of teeth and frequently expose the roots of the teeth due to periodontal damage, severe gingival recession, and alveolar bone loss [14]. Common dental symptoms include teeth loosening and slow healing following tooth extraction [15]. After the breakdown of the alveolar bone, the lesion may produce a dull, constant discomfort, enlargement of the jawbone, soft tissue mass, pain, and ulceration [1]. The initial lytic process of the lesion is tiny, centrally situated, and has irregular, poorly defined boundaries. In the midphase, the boundaries usually get more clearly defined, but in the late phase, periosteal lamellations are resolved, the borders become more well-defined, and occasionally, a thick crust of sclerotic tissue forms. An uncommon clinical symptom is a pathological fracture [4]. A biopsy is advised because the radiographic appearance might resemble malignant tumors [15].

The morphologic, immunohistochemical, and clinical standards necessary for the diagnosis of LCH and other childhood histiocytic illnesses were established by the Writing Group of the Histiocytic Society in 1987. The sequence for the diagnosis of LCH includes the presumptive diagnosis (characteristic light microscopic features), designated diagnosis (light microscopic features plus two or more additional positive stains for S-100 protein, a-o-Mannosidase, peanut lectin, or adenosine triphosphatase), and definitive diagnosis (with electron microscopy, lesional cells with light microscopic properties, and Birbeck granules and/or lesional cells stained positively for CD1a antigen) [3].

The management of LHC depends on the level of involvement. In cases with restricted Langerhans cell histiocytosis involving only skin lesions, at first, therapy might not be required because the lesions frequently resolve on their own, especially in the newborn. Topical steroids are used as the initial line of treatment. If the lesions do not resolve, systemic chemotherapy is typically used to achieve resolution. Patients whose diseases have shown resistance to previous treatments have been treated successfully with topical nitrogen mustard or irradiation [3]. Depending on the location of the illness and the patient's age, low-dose radiation is useful in treating LCH with a total dosage of 3 to 10 Gy. Bone lesions presenting as localized painful lumps are preferably managed by surgical curettage. In cases with isolated lymph node involvement, resolution occurs without therapy [3].

The management of extensive Langerhans cell histiocytosis primarily involves chemotherapy. Due to the progressive nature of generalized LCH, systemic chemotherapeutic drugs have been used to treat the majority of patients. Short-course prednisolone is especially helpful for patients with severe illness but no organ failure since their prognosis is good [3]. According to Kelly and Pritchard, monoclonal antibody treatment is also very effective as a cytotoxin and an immunoregulator [16]. The specificity of a monoclonal antibody directed against the CD1a antigen on Langerhans cells was utilized by these scientists [3]. Patients should be cautiously monitored for a long time since recurrence rates are reported to range from 1.6% to 25% depending on the treatment approach and location of the lesion [17].

## Conclusions

Langerhans cell histiocytosis is an uncommon condition whose cause and pathophysiology are still unknown. The correct understanding of this diverse collection of illnesses may benefit from improvements in immuno-histochemistry, molecular biology, and radiodiagnostic procedures. Given that the disease can be unpredictable, it is important to consider the possibility that the unifocal disease could develop into multifocal. Due to the potential for the condition to worsen if left untreated, it is crucial to include an eosinophilic granuloma in the differential diagnosis of bone lesions in adult subjects. The unpredictable nature of Langerhans cell histiocytosis warrants early management and long-term follow-up.
